# Entrepreneurial action and eudaimonic well-being in a crisis: Insights from entrepreneurs in Sweden during the COVID-19 pandemic

**DOI:** 10.1177/0143831X231154753

**Published:** 2023-02-22

**Authors:** Constanze Eib, Claudia Bernhard-Oettel

**Affiliations:** Department of Psychology, Uppsala University, Sweden; Department of Psychology, Stockholm University, Sweden

**Keywords:** COVID-19, entrepreneurial success, entrepreneurs, well-being, Sweden

## Abstract

Based on transactional stress theory, this article provides an empirical glimpse into how entrepreneurs in Sweden have experienced the COVID-19 pandemic. The authors investigated the impact of two crisis-induced stressors (unpredictability, loneliness) on two aspects of entrepreneurial success (business and personal success) through the indirect effect of eudaimonic well-being. They examined the role of crisis-related entrepreneurial actions (applying for government financial support, engaging in online business activities). Results from a sample of entrepreneurs operating in Sweden in the summer of 2020 revealed that unpredictability and loneliness were negatively related to business and personal success via eudaimonic well-being. Results for the moderating effects of the crisis-related entrepreneurial actions revealed mixed findings. The results provide valuable insights into the mechanisms that tie entrepreneurial stressors and opportunities for action to eudaimonic well-being, and in turn, entrepreneurial success in the early days of the crisis caused by the pandemic.

## Introduction

We want to invite you, the reader, back to the first half of 2020. A global health crisis had just developed, a frightening virus made its way through society. Countries scrambled together a response to save their citizens and the economy. For many, life was put on hold. Offices, shops, schools, cinemas, restaurants closed, travel was no longer possible. And no one knew how long this would continue or what the world would look like after this pandemic ended. In Sweden, there was no formal lockdown, but many people followed the restrictions, kept physical distance and reduced social contacts, and those who had the possibility worked from home to a larger extent (Swedish Agency for Work Environment Expertise, 2021; Trafikanalys, 2022). The security net and labor laws in Sweden are designed to be protective for employees but not for entrepreneurs (Statens Offentliga Utredningar, 2019), and particularly not in a crisis of this magnitude. Many entrepreneurs were faced with fewer customers, the loss of income, the risk of bankruptcy – their ‘business as usual’ was put on hold (Företagarna, 2020). As early as June 2020, a Swedish union warned that six out of 10 entrepreneurs risked bankruptcy as a result of the COVID-19 pandemic (Unionen, 2020). We follow the broad, occupational definition of entrepreneurship ‘as individuals who work for their own account and risk’ (Stephan et al., 2022a: 4), which includes those with a registered business, who may but do not have to employ others.

Entrepreneurs faced a lot of challenges pertaining to unpredictability and social isolation with potential negative effects on their well-being, which is a crucial resource and human capital for entrepreneurs (Torrès and Thurik, 2018). When well-being of entrepreneurs is compromised, success and performance are likely negatively impacted, which threatens the survival of the business (Stephan, 2018). However, studies on entrepreneurs’ well-being are still scarce – despite the calls to change this (Wiklund et al., 2019) – and particularly so during crisis situations. Indeed, evidence from French entrepreneurs suggests that well-being was compromised as burnout levels increased during the COVID-19 pandemic (Torrès et al., 2022). Although many entrepreneurs witnessed that the crisis was detrimental for their businesses, few if any studies have explicitly linked entrepreneurial stressors and well-being to entrepreneurs’ perceptions of success during the crisis. Furthermore, although a number of studies have documented negative developments for entrepreneurs and their businesses, few have shed light onto possible actions to buffer the negative effects. Thus, it is of interest to study what, if anything, entrepreneurs could do to prevent the negative effects of COVID-19-related stressors to their well-being and success.

This study aims to delineate the interrelations of perceived stressors, entrepreneurial well-being and business as well as personal success, and to test whether *crisis-related entrepreneurial actions* (applying for government financial support, engaging in online business activities) had buffering effects. The study took place among Swedish entrepreneurs during the early phase of the COVID-19 pandemic in summer 2020 and makes at least three contributions. First, this study adds to the emerging literature on stressor–strain reactions in entrepreneurs by showing that crisis-induced stressors have important consequences for well-being as well as entrepreneurial success – and that when being confronted by an unprecedented crisis not caused by one’s own actions or failure. Here, we focus on two *crisis-induced stressors*, i.e., stimuli that are experienced as load and trigger strain responses (Lazarus and Folkman, 1984): unpredictability and loneliness. From non-crisis situations, we know that entrepreneurs regularly operate in contexts of unpredictability and also perceive substantial amounts of loneliness (Fernet et al., 2016; Grant and Ferris, 2012; Schonfeld and Mazzola, 2015). However, after COVID-19 was declared a pandemic in March 2020, and many restrictions were put into action, many entrepreneurs had to deal with a sudden drop in clients, quarantine measures, travel bans and lockdowns all around the world, which interrupted or abruptly eroded their usual business opportunities and contacts with clients, suppliers and business partners (Bartik et al., 2020; Grözinger et al., 2022). Entrepreneurs had to tackle unseen levels of isolation and loneliness since there was no one they could turn to for advice, and their usual business routines were not applicable in this unprecedented situation. Moreover, this was a crisis of unforeseeable duration and global magnitude, inducing a large amount of unpredictability into virtually all kinds of businesses. In sum, the COVID-19 pandemic constituted a shock for many entrepreneurs (Bartik et al., 2020; Fairlie, 2020; Kuckertz et al., 2020), which can be expected to have led to immediate negative consequences for their well-being and success.

The study’s second contribution relates to the conceptualization of well-being as a mediator in the relationship between crisis-induced stressors and entrepreneurs’ success. More specifically, we argue that the crisis-induced stressors in terms of unpredictability and loneliness threatened entrepreneurs’ well-being, which, in turn, undermined business and personal success. This follows the argumentation of Torrès and Thurik (2018), viewing entrepreneurs’ well-being as a crucial resource and a substantial human capital. We conceptualize well-being with respect to eudaimonic well-being, since this dimension of well-being has been found to be of particular importance in entrepreneurship (Hahn et al., 2012), but also understudied (Stephan, 2018; Wiklund et al., 2019). Furthermore, we differentiate entrepreneurial success into two aspects: business and personal success. Although business-related and financial success has dominated the literature for a long time (Shepherd et al., 2018; van Praag and Versloot, 2007), entrepreneurs gain more from owning and managing a business than financial aspects can cover (Dijkhuizen et al., 2018; Wach et al., 2016). Hence, studying the aspect of personal success adds to the entrepreneurship literature and follows recent calls for a more elaborate view on relevant outcome variables for entrepreneurs (Wach et al., 2020). With this, we also contribute to the wider literature on business resilience (see Gianiodis et al., 2022) by proposing eudaimonic well-being as a process underlying the relationship between crisis-induced stressors and success.

The third contribution relates both to the entrepreneurship literature but is also of relevant practical importance for policy-makers. Building on the agility framework proposed by Stephan et al. (2022b), this study tests whether crisis-related entrepreneurial actions buffered the negative effects of crisis-induced stressors on eudaimonic well-being, which in turn is associated with business and personal success. Specifically, we focus on two actions: (1) seeking government financial support and (2) engaging in online business activities. Almost all countries offered entrepreneurs and businesses various measures of financial support, such as tax reductions, compensation for income losses, reductions of rental costs, etc. However, the effect of these support options, particularly on stressor–strain relationships, are still understudied and evidence on the positive effects are mixed (Belghitar et al., 2022; Dörr et al., 2022; Tetlow and Dalton, 2020). Moreover, many predicted radical changes in business operations and stronger digitalization efforts as a result of the pandemic (Kuckertz et al., 2020; Zahra, 2021). Here, we study whether engaging in online business activities, such as home delivery of food by restaurants, online teaching and e-commerce, can mitigate the negative effects of the crisis-induced stressors on well-being, and in turn, business and personal success.

## Theory and hypotheses

### Crisis-induced stressors and well-being

One of the most influential psychosocial stress theories is the transactional stress theory by Lazarus and Folkman (1984), which proposes two appraisal processes when individuals perceive potentially threatening events or situations. During the primary appraisal process, individuals make an interpretation as to whether an event or situation is irrelevant, positive or threatening. If individuals conclude that the situation is threatening, they undergo a secondary appraisal process, in which they make an assessment as to whether they have sufficient resources to cope with the stressor. When individuals conclude that they can overcome the stressor, the likelihood a rises that problem-coping strategies are activated to focus on dealing with the problem head-on. When individuals appraise that they cannot overcome that stressor, the likelihood is higher that individuals engage in emotion-focused coping, which may include withdrawal, distancing, venting, or attempts to avoid the stressor. Either way, evidence suggests that threat appraisals result in individuals developing strain, such as depression, exhaustion, or health complaints (LePine et al., 2005; Podsakoff et al., 2007).

**Figure 1.**
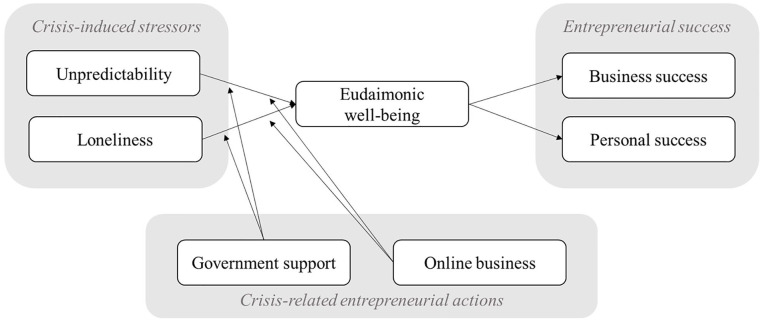
Hypothesized model.

Based on the theoretical framework of transactional stress theory, we will in the following elucidate how the stressors unpredictability and loneliness during the COVID-19 pandemic may relate to eudaimonic well-being and in turn, business and personal success, and how crisis-related entrepreneurial actions (seeking government financial support, online business activities) may buffer the negative indirect effects of the stressors via well-being to success. Figure 1 illustrates the conceptual model of this article. The hypotheses are explained in the following paragraphs.

#### Unpredictability

Unpredictability is inherent in entrepreneurship (McMullen and Shepherd, 2006) and previous studies show that unpredictability is associated negatively with entrepreneurial well-being (Stephan, 2018). This is also in line with transactional stress theory, as unpredictability is likely appraised as threatening, eliciting an assessment of whether resources are available to tackle the stressor. However, with the COVID-19 pandemic coming as a shock for many business owners, unpredictability became an all-pervasive stressor (Sinclair et al., 2021). The economic crisis, fear of losing the business and income, the inability to make long-term plans or investments were all challenges that were likely perceived as threatening for entrepreneurs (Rigotti et al., 2021). During COVID-19, many aspects of the external environment changed rapidly; for instance, new regulations and recommendations in Sweden were often updated only at short notice. Particularly in the beginning of the pandemic, it was very uncertain whether businesses would be allowed to remain open, whether customers would be willing or able to pay, whether there would be supportive measures in place for businesses, or how long the crisis would last. Moreover, the pandemic has also introduced profound and continuous changes for clients and for society as a whole, which made it difficult to predict how the future would look like after the pandemic, and thus, planning ahead became very difficult. Thus, we argue that entrepreneurial unpredictability in light of the COVID-19 pandemic is a stressor that undermines entrepreneurs’ eudaimonic well-being.

#### Loneliness

Loneliness or isolation is a common stressor for entrepreneurs (Grant and Ferris, 2012). Compared to managers or employees, entrepreneurs lack an organizational network, supervisors or colleagues. Even confiding with other business owners may be problematic as these others may be or turn into competitors (Schonfeld and Mazzola, 2015). Most entrepreneurs operate in very small businesses, alone or with few employees or business associates (Boyd and Gumpert, 1983; European Commission, 2018). During normal work days, for many entrepreneurs, their social contact may, to a large extent, stem from meeting their customers.

Loneliness is commonly described as a negative phenomenon, as an individual’s perception that human connection is lacking, as an unpleasant state, in which social needs are not met (de Jong Gierveld, 1998). Various coping strategies have been suggested for loneliness (Boyd and Gumpert, 1983), which signals that loneliness can be considered a stressor that elicits secondary appraisals in line with transactional stress theory. With the sudden onset of a pandemic that no one in Sweden had experienced in their lifetime before, there was also no one entrepreneurs could turn to for guidance or advice. While there was no strict lockdown in Sweden, regulations were still put in place to reduce physical contact with others, so that meeting an employee, a business associate, and most of all, the different customers was kept to a minimum or was practically impossible. This most likely has exacerbated levels of entrepreneurial loneliness. Evidence from French entrepreneurs suggests that loneliness during COVID-19 was associated with higher levels of burnout (Torrès et al., 2022). A study from Brazil found that it was loneliness and not social distancing that was associated with more suicidal tendencies (Antonelli-Salgado et al., 2021). Also, studies before COVID-19 showed that loneliness is associated negatively with entrepreneurs’ well-being (Stephan, 2018). We thus expect experiencing loneliness during the pandemic is negatively associated with entrepreneurs’ well-being.

### Well-being and entrepreneurial success during a crisis

The literature on the association between well-being and entrepreneurial performance and success is surprisingly scarce (Gorgievski and Stephan, 2016). There is some empirical evidence that positive and negative affect, two indicators of hedonic well-being, are associated with business effort (Foo et al., 2009). A longitudinal study showed that well-being, with the indicators job satisfaction, life satisfaction and engagement, predicted both subjective financial success and personal success of entrepreneurs (Dijkhuizen et al., 2018). The authors suggested that their study provides evidence for the happy-productive hypothesis among entrepreneurs, such that satisfied and engaged entrepreneurs also have more resources to be productive and perform well. Other scholars have argued that well-being is beneficial for effective information processing and decision-making, and thus, facilitates performance (Gorgievski et al., 2010).

A recent study showed that it was eudaimonic well-being but not hedonic well-being that predicted proactivity (Hahn et al., 2012), which is important for entrepreneurs who constantly have to initiate actions to keep the business running successfully. Similarly, factors associated with eudaimonic well-being, such as the ability to cope with challenges, have been found to relate to higher persistence in entrepreneurship (Patel and Thatcher, 2014). Eudaimonic well-being is a component of well-being that relates to having a purpose in life, fulfilling one’s potential and aspirations, perceiving control in life as well as accepting one’s strengths and weaknesses and having quality relationships with others (Culbertson et al., 2010; Ryff, 2019). Ryff (2019) proposed that eudaimonic well-being may be a critical psychological resource that can contribute to the success of entrepreneurial businesses. This implies that eudaimonic well-being may be of relevance to entrepreneurial success, particularly in a crisis such as the COVID-19 pandemic that requires persistence, initiative and proactivity to keep the business running successfully. However, the association between eudaimonic well-being and performance or success has rarely been empirically examined. These ideas on the relationship between well-being and performance are consistent with transactional stress theory, such that lack of well-being is an indicator of strain, which can undermine the effective functioning of individuals (Lazarus and Folkman, 1984). Particularly, this may be the case in a crisis such as the pandemic, in which well-being may have been compromised for many entrepreneurs, and business success was extra difficult to reach, given the much-changed situation. Based on transactional stress theory and the existing empirical evidence, we suggest the stressors unpredictability and loneliness relate to reduced eudaimonic well-being, which, in turn, associates with less business and personal success. In other words, we propose indirect relations in a mediation framework and our hypotheses read as follows:

*H1: There is an indirect effect between unpredictability and (a) business success, (b) personal success via eudaimonic well-being.*
*H2: There is an indirect effect between loneliness and (a) business success, (b) personal success via eudaimonic well-being.*

### Crisis-related entrepreneurial actions

A relevant lesson from the 2008 financial crisis was that many companies risked bankruptcy (Bernhard-Oettel et al., 2019; Fairlie, 2013), but that certain companies adapted to the new circumstances and survived the crisis. Similarly, there is evidence that, for example, small business owners who reacted quickly to the demands of the New Zealand earthquake disaster had better chances to be successful after the crisis (Battisti and Deakins, 2017). There is anecdotal evidence that in light of the COVID-19 restrictions and regulations, many companies attempted to adapt to the crisis by changing products, attracting new customers, or shifting production focus (Zahra, 2021). At the same time, it was also noted that innovations during the beginning of the pandemic were difficult at least for small or new businesses as most investors, customers and business partners were themselves heavily engaged in adapting to the pandemic (Kuckertz et al., 2020). The limited available empirical evidence suggests small-scale adaptations rather than revolutionary changes to business models, markets and customers (Fasth and Elliot, 2021).

In a recent paper, Stephan et al. (2022b) proposed entrepreneurial agility as a mechanism for business owners to counter adversity. They defined agility as adaptive actions to adapt to environmental changes. Entrepreneurs high on agility are those that ‘act’ and engage in flexible and adaptive responses whereas those low on agility rather opt for a ‘wait-and-see’ strategy. The authors argue that agility actions give entrepreneurs some of the control back that they miss due to the adversity faced, may increase their well-being and alleviate uncertainty or help find new income sources. However, Stephan et al. (2022b) investigated the effect of changing business plans and seeing new business opportunities as agile actions, which are limited in their specificity. Instead, in this current study, among the possible agility actions entrepreneurs can take, we selected two to investigate further: *seeking financial government support* and *engaging in online business activities*.

#### Seeking financial government support

In order to save businesses from insolvency, many governments reacted to the COVID-19 pandemic by offering various support schemes (Tetlow and Dalton, 2020). The support schemes varied a lot by country, by magnitude of the available support, and type of support. It has been estimated that in the European Union support for distressed sectors increased by 6 percentage points in 2020 compared to before the pandemic (Dörr et al., 2022). In Sweden, the situation looked slightly different. In a comparative review of the support schemes in several Western countries, Tetlow and Dalton (2020) found that the Swedish government had guaranteed only 0.05% of the GDP up until mid-September 2020, which was the lowest percentage point of all countries compared (including Canada, the UK, Norway, France, New Zealand, Germany, etc.).

From a psychological perspective, applying for government support schemes can be seen as an active and agile action for entrepreneurs (Stephan et al., 2022b) and resembles a problem-focused coping strategy (Lazarus and Folkman, 1984). It means taking the opportunity to potentially increase funding and cash flow, which helps to maneuver adversity and keep the business afloat. It may reduce the worries about finance and the future induced by unpredictably and thus may protect entrepreneurial well-being. Seeking government support may also be perceived as trying to take back control, since entrepreneurs may feel that they are active and doing all they can to reduce unpredictability. Regarding loneliness, seeking government support may also be of help to reduce the negative effects to well-being. The fact that government support options were made available can signify two different positive points that may buffer the negative effects of loneliness. First, entrepreneurs may feel they and their struggle are acknowledged and are deemed important enough to warrant support. Second, it may also signal to entrepreneurs that others are struggling as well and that it is the environment that is challenging their business and not their capacity to do business. Thus, we predict:

*H3: The indirect effect between unpredictability and (a) business success and (b) personal success via eudaimonic well-being is moderated by seeking government support, such that the indirect effect is weaker for those that have applied for government funding.*
*H4: The indirect effect between loneliness and (a) business success and (b) personal success via eudaimonic well-being is moderated by seeking government support, such that the indirect effect is weaker for those that have applied for government funding.*

#### Engaging in online business activities

Similar to the agility approach proposed by Stephan et al. (2022b), Wernberg (2020) commented on the reality for businesses as a result of the early stages of the COVID-19 pandemic: ‘The combination of uncertainty and slowness [of the pandemic] means that . . . each company must decide whether to hold its breath while waiting to be able to return to regular operations or try to adapt its business operations to the supposed new normal state after the pandemic’ (p. 13). Further, Wernberg (2020) writes that many Swedish companies were forced to use digital tools as substitutes for offices, meeting places and physical meetings. Likewise, it was noted that digital technology was one of the major changes brought about by the COVID-19 pandemic but also was highlighted to be a viable new business opportunity during the pandemic (Kuckertz et al., 2020; Zahra, 2021). Digitalization and introducing digital tools and working procedures were already a major development for businesses prior to the pandemic (Tillväxtverket, 2018; see also Zahra, 2021). There is some tentative support that digitalization efforts had positive effects for businesses during the pandemic (Audretsch and Belitski, 2021; Priyono et al., 2020).

Psychologically, engaging in online business activities may also be seen as an active problem-solving strategy of adapting to the situation and trying to solve the problems of not being able to meet customers or business partners in person, and instead, opting for reaching customers and markets through digital means. Engaging in online business activities may buffer the negative effects of unpredictability on eudaimonic well-being for several reasons. As it was unclear how long the pandemic would continue, engaging in online business activities may be one option to keep the business afloat until the pandemic is over. Engaging in online business activities is also a way to interact with customers that the entrepreneur may otherwise not meet. As an additional plus, entrepreneurs can reach new customers through digital means; they can also create new opportunities and learn new skills relevant for the future. Yet another effect of engaging in online business activities is having less time to worry about the current situation. In sum, we expect that entrepreneurs engaging in online business activities may feel more in control and may also learn new skills or perceive themselves as being agile, which should help overcome the stressors of the pandemic unpredictability and loneliness. Thus, we predict:

*H5: The indirect effect between unpredictability and (a) business success and (b) personal success via eudaimonic well-being is moderated by online business activities, such that the indirect effect is weaker for those that have engaged in online business activities.*
*H6: The indirect effect between loneliness and (a) business success and (b) personal success via eudaimonic well-being is moderated by online business activities, such that the indirect effect is weaker for those that have engaged in online business activities.*

## Methods

### Context

The first case of COVID-19 in Sweden was confirmed at the end of January 2020. In the second week of March 2020, the Public Health Agency recommended that residents keep physical distance from others, some types of schools and universities were to move to online teaching and workers should work from home if possible (Hensvik and Nordström Skans, 2020). Gatherings were limited to 50 people, outdoor activities were allowed if physical distance could be guaranteed, and shops and businesses remained open. Sweden did not introduce a lockdown at any point during the pandemic but recommendations by the Public Health Agency had sharp effects on businesses. All sectors lost in sales compared to previous years, with a particularly high level of sales loss between March until summer (Swedish Fiscal Policy Council, 2021). Some sectors were hit particularly hard, for instance, sectors like restaurants, hotels and travel-related businesses dropped 70% of their sales. The unemployment rate rose, layoffs increased (more so than during the 2008 financial crisis) and the number of vacancies dropped sharply (Swedish Fiscal Policy Council, 2021).

In 2020, most of the financial government support in Sweden was paid out between the end of March until June (Swedish Fiscal Policy Council, 2021). During the first months of the pandemic in 2020, several types of support schemes were available to businesses. One type was tax payment deferrals, meaning that businesses were allowed to pay taxes at a later stage. This type of support helps businesses maintain liquidity for a few months but only postpones payments. Particularly medium-sized businesses used this support scheme. Another type was temporarily reduced employer and personal contribution costs for the months between March and June 2020. Another type was furloughing employees, that is, businesses with employees could reduce their labor costs substantially. The government shared the costs associated with having employees with the business owner while employees reduced their work time but retained a large part of their salary. This scheme was announced in April and continued well into 2021. More than 500,000 workers were affected at some point during 2020 by this scheme. There was also a restructuring support program which was introduced to compensate for part of the losses in sales. Sales loss was compared to the same period in 2019, had to be related to COVID-19, and had to be higher than a certain percentage (between 30 and 50% depending on the timing during the pandemic) and businesses had to have a certain revenue in the past year. In practice, businesses did not apply for this type of support to the extent that the government expected (8% of the expected value was paid out until early 2021). One of the reasons was that most businesses lost up to 30% but not more in sales after the initial first months of the pandemic in 2020. Another reason was that the administrative burden for this type of support was high. Another support scheme involved that landlords could get some compensation for renting out space to businesses in certain sectors between April and June 2020. An evaluation of the support schemes by a so-called Corona commission in 2022 (Statens Offentliga Utredningar, 2022) concluded that a substantial amount of the money paid to businesses went to businesses who actually increased their revenue during the pandemic. That is, businesses were supported that did not need any help. That was possible because the support schemes were not all formulated to target only those businesses that needed help. Moreover, the commission noted that some business owners did not apply for any support schemes, and others waited for decisions on their applications for months only to get a faulty decision they subsequently appealed.

One major caveat of the support schemes is that, for a long time during 2020, they only considered businesses of a certain company type. The most common company type is sole proprietorship companies, and many of the entrepreneurs with this company type get the majority of their income from their company. Sole proprietorship company owners mostly did not qualify for the restructuring support program (this program was modified to fit this group in November 2020), and they could not use the furloughing scheme because they are not employed in their companies. They could also not enlist as unemployed because the company, in effect, would need to be put into liquidation. However, they were allowed to use the schemes for tax payment deferrals and temporarily reduced employer and personal contribution costs (Swedish Fiscal Policy Council, 2021) but not all did so (Statens Offentliga Utredningar, 2022).

### Data collection

Data stem from a larger project that examined how the COVID-19 pandemic affects entrepreneurs’ work environment, well-being and health in Sweden (Eib and Bernhard-Oettel, 2020). The project is part of a global research initiative encompassing 28 countries organized by King’s College London. The Swedish part of the data collection received ethical approval by the Swedish Ethical Review Board (2020-02467).

### Participants

Participants were recruited through contacts with entrepreneurial interest groups and through various websites and social media channels. Informed consent was collected from all participants. The data collection occurred between May and July 2020, thus during an intensive period of the COVID-19 pandemic in Sweden.

In total, 216 entrepreneurs started the questionnaire; 42 of these did not provide any answers on any of the study variables and were therefore excluded. Of the remaining 174 participants, 60% of the entrepreneurs were women, had an average age of 51 years (SD = 9.35, range = 29–78), around 20% had no university education, 27% a university education with a maximum of three years, and around 53% a university education of at least four years. In terms of sector, 40% classified their work into business services (e.g., consultancy, legal advice, software development), 30% into human-facing services (e.g., healthcare, social services, education), 18% into culture, arts and design, and 13% into retail, hotel and gastronomy. Concerning age of business, around 6% worked in businesses that were started in 1990 or earlier, 17% were started between 1991 and 2000, 29% were started between 2001 and 2010, 21% were started between 2011 and 2015, and the remaining 27% were started between 2016 and 2020 (but before the pandemic started).

### Measures

#### Unpredictability

Unpredictability was measured with four items based on Grant and Ferris (2012). The items were: ‘I face uncertainty about the future of my business’, ‘I have to deal with unforeseen problems at work’, ‘I have to make decision(s) where I am unsure about their effects on my business’, ‘My entrepreneurial work seems uncertain and risky’ with an answer scale ranging from 1 (*strongly disagree*) to 5 (*strongly agree*). Cronbach’s alpha was .815.

#### Loneliness

Loneliness was assessed with three items based on Grant and Ferris (2012). Items were ‘At work, I feel lonely and alone’, ‘There is not much social support from people I interact with at work’, ‘There is no one at work with whom I can share experiences or new ideas’ with an answer scale ranging between 1 (*strongly disagree*) to 5 (*strongly agree*). Entrepreneurs were instructed to answer these questions in relation to the last 30 days. Cronbach’s alpha for was .751.

#### Seeking government support

Seeking financial government support was assessed with the question ‘Have you applied for government support (e.g. furloughing workers, business loan)? Select all options that apply.’ The question was followed with different answer options. Eight of the options referred to different support schemes offered at the time of the data collection to businesses operating in Sweden. These included options such as business loans, turnover support, support regarding furloughing employees, deferrals of taxes, rental support, etc. and an ‘other’ option where entrepreneurs could enter any other support option they had applied for. The last option was ‘I did not apply for any government support’. The variable was then coded in a way so that ‘0’ refers to those that have not applied for any support option versus ‘1’ referring to those that have applied for one or several support options.

#### Online business

Engaging in online business activities was assessed with one question: ‘Did you newly expand your business into online trading or delivery services?’ with the answer options 1 (*yes*), 2 (*no*) and 3 (*online trading and delivery already existed*). The variable was recoded so that ‘0’ refers to those that have answered ‘no’, and ‘1’ refers to those that have answered ‘yes’ or ‘online trading and delivery already existed’.

#### Eudaimonic well-being

Based on Culbertson et al. (2010), six indicators were used to assess well-being. An example item was: ‘I feel positive about myself and the events that happened at work’. The answer scale ranged from 1 (*strongly disagree*) to 5 (*strongly agree*). Cronbach’s alpha for the six items was .681. Excluding the item ‘The expectations of others affect my actions and my thinking about my work’ increased the alpha level to .771. An exploratory factor analysis extracted one factor of the remaining five items, with good communalities. A factor analysis with six items showed a preference for a two-factor solution, with the item we decided to exclude showing problematic negative loadings. Thus, we decided to calculate a mean score of the five items, with higher levels indicating more eudaimonic well-being.

#### Personal success

Personal success was assessed with the personal fulfillment aspect as outlined by Wach et al. (2020). All three items were introduced with ‘How successful have you been in the past month in achieving the following aspects in your business?’ The items concerned personal work flexibility, own decision-making and personal development, and the answer scale ranged between 1 (*not achieved at all*) to 5 (*absolutely achieved*). Cronbach’s alpha was .772.

#### Business success

Business success was measured with three items based on Wach et al. (2020) and Yu et al. (2019), and all items were introduced with ‘How successful is your business currently? Please rate how successful your business has been during the last month compared to competitors concerning . . .’) and the questions concerned profit, sales development and cash flow. The scale ranged between 1 (*much worse than the competition*) and 5 (*much better than the competition*). Cronbach’s alpha was .939.

### Analytical strategy

Given the sample size, we decided to use path models, where variables are entered as observed or manifest variables, instead of latent variables (Bollen, 1989). Hypotheses 1 and 2 were about the indirect effects of eudaimonic well-being for the relationships between predictability and loneliness and business and personal success. A path model was specified that included the two independent variables (predictability, loneliness), the mediator (eudaimonic well-being) and the two dependent variables (business and personal success). Confidence intervals were generated by bootstrap procedures with 5,000 samples.

Hypotheses 3–6 were about the conditional indirect effects. To reduce model complexity given the sample size, the interaction effects were tested separately. Specifically, one path model included the interaction effects of the independent variables with seeking financial government support and another path model included the interaction effects with engaging in online business activities. To test indirect and conditional indirect effects, significance and confidence intervals generated by bootstrap procedures with 5,000 samples were examined. Analyses were carried out with Mplus 8 (Muthén and Muthén, 2017), with full information maximum likelihood (FIML) used to reduce bias due to missing data (Enders and Bandalos, 2001). We also ran the analysis when controlling for age of business, business form, education of entrepreneur, founder status, having employees or not and gender, and conclusions remained the same. To limit the addition of variables not included in our hypotheses, we only display results without covariates. In order to further test for robustness of results, we created new continuous variables of business success and personal success variables by only including scores at the 25% and 75% percentile of each variable, respectively. Analyses based on these extreme cases remained almost identical.

## Results

Table 1 shows the descriptive statistics and correlations among the study variables. As expected, unpredictability is negatively associated with well-being, business and personal success. Loneliness, however, was only significantly associated with less well-being and personal success but unrelated to business success. Eudaimonic well-being was positively associated with business and personal success. Having applied for financial government support was positively associated with unpredictability, negatively associated with well-being but was not significantly correlated with business or personal success. Having engaged in some form of online business activities was positively associated with well-being. Eudaimonic well-being and personal success were substantially correlated (*r* = .51, *p* < .001) but not as high as to suggest that they are the same constructs. Thus, while a significant path estimate between well-being and personal success is likely, indirect and conditional indirect effects may or may not be there. Finally, while business and personal success were positively correlated, the correlation of *r* = .30 (*p* < .001) suggests that the constructs are sufficiently separate to warrant that results can differ between them.

**Table 1.** Means, standard deviations and correlations of study variables.

	Variable	M	SD	1	2	3	4	5	6	7
1	Unpredictability	3.60	0.99	1						
2	Loneliness	2.68	1.08	.14	1					
3	Eudaimonic well-being	3.85	0.79	−.35***	−.38***	1				
4	Business success	2.94	0.86	−.25**	−.10	.37***	1			
5	Personal success	3.13	1.17	−.31***	−.22**	.51***	.30***	1		
6	Government support	0.46	0.50	.24**	−.10	−.19*	−.05	−.13	1	
7	Online business	0.29	0.45	.10	−.09	.19*	.02	.01	−.07	1

*Note. N* = 174. ***p < .001, **p < .01, *p < .05.

Hypotheses H1 and H2 stated that there are indirect effects between unpredictability and loneliness and business and personal success through the effect of eudaimonic well-being. Results displayed in Table 2 show that the indirect effect of unpredictability to business success via eudaimonic well-being was significantly negative (a*b = −.089; 95%CI −.153; −.024), with unpredictability being negatively related to eudaimonic well-being (β = −.31, *p <* .001) and well-being positively related to business success (β =.33, *p <* .001). Thus, H1a was supported. Similarly, the indirect effect between unpredictability and personal success was also significantly negative (a*b = −.162; 95%CI −.257; −.068), with eudaimonic well-being positively related to personal success (β =.45, *p <* .001). Thus, H1b was also supported. The indirect effect between loneliness and business success was significantly negative (a*b = −.093; 95%CI −.160; −.025), with loneliness negatively related to eudaimonic well-being (β = −.35, *p <* .001). Thus, H2a was supported. Similarly, the indirect effect between loneliness and personal success was also significantly negative (a*b = −.170; 95%CI −.263; −.077). Thus, H2b was also supported.

**Table 2.** Test of indirect effects H1–H2.

	Business success	Personal success
	β (*p*)	β (*p*)
Unpredictability to EWB	−.31 (< .001)	
Loneliness to EWB	−.35 (< .001)	
EWB to DV	.33 (< .001)	.45 (< .001)
Unpredictability to DV	−.14 (.087)	−.15 (.046)
Loneliness to DV	.04 (.656)	−.03 (.713)
*Model fit*	χ^2^ = 3.23 (df = 1), *p* = .072, CFI .981, RMSEA .113, SRMSR .041	
*Indirect effect unpredictability*	−.089, *p* = .007 (95%CI −.153; −.024)	−.162, *p* = .001 (95%CI −.257; −.068)
*Indirect effect loneliness*	−.093, *p* = .007 (95%CI −.160; −.025)	−.170, *p* < .001 (95%CI −.263; −.077)

*Note. N* = 174. EWB = eudaimonic well-being. DV = dependent variable. CFI = comparative fit index. RMSEA = root mean square error of approximation. SRMSR = standardized root mean square residual. Variance explanation *R*^2^ (business success: .148; personal success: .272; EWB: .220).

Hypotheses 3 and 4 stated the expectations that the indirect effects between unpredictability and business and personal success were conditional upon seeking financial government support. Table 3 displays the estimates for the models including the interaction effects, and Table 4 shows the results of the moderated mediation bootstrap results. Variance explanation was .22 for business success, .34 for personal success and varied for eudaimonic well-being between .29 and .32.

**Table 3.** Test of conditional indirect effects H3–H6.

	Business success	Personal success	Business success	Personal success
	β (*p*)	β (*p*)	β (*p*)	β (*p*)
Unpredictability to EWB	−.31 (< .001)		−.26 (< .001)	
Loneliness to EWB	−.17 (.070)		−.39 (< .001)	
Government support to EWB	−.16 (.025)		−.15 (.041)	
Online business to EWB	.16 (.025)		.18 (.009)	
Unpredictability × government support to EWB	.04 (.616)		–	
Loneliness × government support to EWB	−.26 (.012)		–	
Unpredictability × online business to EWB	–		−.05 (.586)	
Loneliness × online business to EWB	–		.07 (.422)	
EWB to DV	.32 (< .001)	.43 (< .001)	.32 (< .001)	.43 (< .001)
Unpredictability to DV	−.15 (.079)	−.15 (.041)	−.15 (.079)	−.15 (.041)
Loneliness to DV	.06 (.517)	−.02 (.795)	.06 (.517)	−.02 (.795)
*Model fit*	χ^2^ = 5.58 (df = 8), *p* = .694, SRMSR .024		χ^2^ = 3.52 (df = 8), *p* = .898, SRMSR .022	

*Note*. N = *153*. EWB = eudaimonic well-being. DV = dependent variable. SRMSR = standardized root mean square residual. Variance explanation *R*^2^ (business success: .142; personal success: .259; EWB: .294 in Model including online business and .323 in Model including government support).

**Table 4.** Conditional indirect effects H3–H6.

	Business success			Personal success		
	Estimate (*p*)	LL95%CI	UL95%CI	Estimate (*p*)	LL95%CI	UL95%CI
*Conditional indirect effect unpredictability*						
Not applied for government support	−.102 (.113)	−.229	.024	−.183 (.087)	−.393	.026
Applied for government support	−.066 (.080)	−.140	.008	**−.118 (.047)**	**−.234**	**−.002**
*Conditional indirect effect loneliness*						
Not applied for government support	.055 (.419)	−.079	.189	.099 (.393)	−.128	.325
Applied for government support	**−.149 (.009)**	**−.262**	**−.037**	**−.268 (.001)**	**−.422**	**−.114**
*Conditional indirect effect unpredictability*						
Not engaged in online business	−.107 (.092)	−.232	.018	**−.192 (.043)**	**−.378**	**−.006**
Engaged in online business	−.038 (.456)	−.136	.061	−.067 (.450)	−.242	.107
*Conditional indirect effect loneliness*						
Not engaged in online business	−.080 (.206)	−.204	.044	−.143 (.154)	−.341	.054
Engaged in online business	−.132 (.063)	−.272	.007	**−.237 (.043)**	**−.466**	**−.008**

*Note. N* = 153. LL = lower level. UL = upper level. CI = confidence interval. Significant estimates are indicated in bold.

The conditional indirect effect of unpredictability on business success was not significant for entrepreneurs seeking government support (−.066; 95%CI −.140; .008) or not (−.102; 95%CI −.229; .024), thus H3a was not supported. One can note, however, that the indirect effect without taking into consideration the moderator seeking government support was a*b = −.089. Thus, there is some tendency that seeking government support changes the indirect effect in different directions: with seeking government support, the indirect effect is less negative; without government support, the indirect effect is slightly more negative.

The conditional indirect effect of unpredictability on personal success was not significant (−.183; 95%CI −.393; .026) for those who did not seek government support, but was significantly negative (−.118; 95%CI −.234; −.002) for those who sought government support. This result was against the hypothesis, thus H3b was not supported. Equally here, it is important to note that the indirect effect without the moderator was a*b = −.162. Having sought government support made this indirect effect less negative, and not having sought government support made the indirect effect more negative, although the confidence intervals do include zero.

Regarding H4a, the conditional indirect effect between loneliness and business success was not significant (.055; 95%CI −.079; .189) for those who did not seek government support, whereas it was significantly negative (−.149; 95%CI −.262; −.037) for those who sought government support. This result was against the hypothesis, thus H4a was not supported. Similarly, the conditional indirect effect between loneliness and personal success was not significant (.099; 95%CI −.128; .325) for those who did not seek government support, whereas it was significantly negative (−.268; 95%CI −.422; −.114) for those who sought government support. This result was against the hypothesis, thus H4b was not supported.

Hypotheses 5 and 6 related to the influence of engaging in online business activities for the indirect effects. The conditional indirect effect of unpredictability on business success was not significantly different dependent on the moderator of engaging in online business activities, thus H5a was not supported. One can note, however, that the indirect effect without taking into consideration the moderator online business activities was a*b = −.089. Thus, there is some tendency that engaging in online business activities changes the indirect effect in different directions: with online business activities, the indirect effect is less negative; when not engaging in online business activities, the indirect effect is slightly more negative. The conditional indirect effect of unpredictability on personal success was not significant for those that engaged in online business activities (−.067; 95%CI −.242; .107) but was significantly negative for those that did not (−.192; 95%CI −378; −.006). This supports Hypothesis H5b.

Regarding H6a, the indirect effect of loneliness on business success through eudaimonic well-being was not different for those who engaged in online business activities (−.132; 95%CI −.272; .007) or not (−.080; 95%CI −.204; 044), thus H6a was not supported. Lastly, the indirect effect of loneliness on personal success was significantly negative for those who engaged in online business activities (−.237; 95%CI −.466; −.008) but not significant for those who did not engage in online business activities (−.143; 95%CI -.341; .054), meaning H6b is not supported.

## Discussion

This study aimed to investigate the role of unpredictability and loneliness for entrepreneurs operating in Sweden during the COVID-19 pandemic as predictors of eudaimonic well-being, which, in turn, was hypothesized to be associated with business and personal success. It was also studied whether two crisis-related entrepreneurial actions (applying for government financial support, engaging in online business activities) would buffer the relationship between the crisis-induced stressors and eudaimonic well-being. Results revealed that unpredictability and loneliness were associated to business and personal success via eudaimonic well-being. The results of the two crisis-related business interventions were mixed and are discussed below.

### Crisis-induced stressors, eudaimonic well-being and entrepreneurial success

Clearly, Swedish entrepreneurs in this study faced unpredictability, and given the descriptive results, this stressor was somewhat more of a concern than the other crisis-induced stressor included in this study, namely loneliness. Associations between crisis-induced stressors and eudaimonic well-being as well as associations between eudaimonic well-being and entrepreneurial success were substantial and significant. Thus, our first two hypotheses on the indirect effects of unpredictability and loneliness on success via eudaimonic well-being found support. The results are consistent with the transactional stress theory (Lazarus and Folkman, 1984): unpredictability and also loneliness are likely experienced as stressors that tax coping mechanisms. The fact that unpredictability was also negatively correlated with business success (*r* = −.25, *p* = .002) emphasizes the stressor–strain perspective. Contrary to beliefs that entrepreneurs thrive under pressure and uncertainty (Baron et al., 2016), our results suggest that this may not be the case. Unpredictability, at least that experienced during the COVID-19 pandemic, undermined not only business success but also eudaimonic well-being and personal success.

Eudaimonic well-being has attracted increasing research interest among entrepreneurial scholars (Ryff, 2019), but very few studies have empirically investigated eudaimonic well-being (for an overview, see Stephan, 2018) and perhaps no earlier study has scrutinized its relations to different success measures during a profound crisis. Here, clearly, this study fills a gap and contributes to the literature by showing that eudaimonic well-being is an important aspect to better understand the mechanisms that tie stressors to performance.

Following recent calls to research different aspects of success in more detail (Wach et al., 2016), we investigated two types of entrepreneurial success – business success and personal success. Few studies so far have considered both aspects of success in the same study. Personal success and business success were significantly but not very highly correlated with one another (*r* = .30, *p <* .001). Furthermore, the associations from the two stressors and eudaimonic well-being differed for both aspects of success, providing further evidence that business and personal success can, in part, be achieved in different ways. We also found that the stressors were indirectly, via eudaimonic well-being, more consistently related to personal success, whereas for business success, the indirect paths had smaller effect sizes. However, it has to be kept in mind that these constructs are complex and are determined by multiple predictors. Further, there is no established metric to evaluate the size of indirect or conditional indirect effects (Preacher and Kelley, 2011). Thus, in field studies, small effect sizes are to be expected (Frese and Zapf, 1988). This result provides further evidence that various aspects of success are important to entrepreneurs, and that financial success is only one part of the picture (Wach et al., 2016). Future studies may expand the scope of this line of research and include further aspects of personal success, for example by including other non-financial success factors that entrepreneurs may find personally rewarding, such as researching community impact (Wach et al., 2016).

### Entrepreneurial actions

In this study, we tested four conditional indirect effects, that, in part, were significant. However, the pattern of results is more complex than initially proposed in the hypotheses. Concerning the application for government support schemes, we found some significant conditional indirect effects for the association between loneliness, eudaimonic well-being and business as well as personal success. However, in contrast to our expectations, the indirect effects were not buffered but accentuated for those who had applied for government support. For unpredictability, the idea that those who applied for support had protected their well-being and, in turn, their success was supported by the data when it comes to personal success, but for business success, applying for government support did not make any difference. There are several conceivable explanations for these somewhat surprising findings regarding government support. First, perhaps the theoretical notion that applying for support induces some sort of control may be more relevant when the outcome is perceived personal success. Here, indeed, having done what was possible may induce a feeling of being agentic and proactive and create a sense of personal mastery, which, in turn, may translate into feeling personally successful in difficult times. However, one major explanation for the fact that applying for government support did not make any difference for business success may be that applying may not be the same as indeed getting financial support. When the data were collected, most entrepreneurs had not yet received a decision on their application, which may have restricted their sense of control and any feelings of relief against income shortage. Furthermore, the Confederation of Swedish Enterprises criticized the government support schemes on several fronts: that the uptake of government funds had been slow, waiting times for applications and payment very long, that appealing against negative decisions took a very long time, and that support was needed much more immediately (Confederation of Swedish Enterprises, 2021). Our study adds to this picture from a psychological angle, as it shows that those who applied, contrary to our expectation, had more pronounced effects from loneliness on eudaimonic well-being and success, instead of, as we had expected, attenuated effects. Furthermore, screening the comments given in open answer formats in the questionnaire, we found that some entrepreneurs did not apply for funds because this would include bank loans, which was seen as putting their financial future at risk, and others did not qualify for any funds at all (Eib and Bernhard-Oettel, 2020). This illustrates that those entrepreneurs who did not apply for government support schemes may have consisted both of entrepreneurs who did not need financial support at any price, and those who may have needed it but were not eligible to apply for support. Furthermore, we conducted a *t*-test with government support as a categorial variable and business success as a criterion variable. Results revealed a non-significant difference (*t*(160) = .62, *p* = .537), suggesting that business success was similar for those who did seek government support (M = 2.91, SD = .82) and those that did not apply (M = 2.99, SD = .89). In sum, it appears that government support schemes in Sweden were insufficient and did not help entrepreneurs protect their well-being when faced with crisis-induced stressors.

Considering the role of engaging in online business activities as an entrepreneurial action, the findings were also more complex than initially expected. Without online business activities, the association of unpredictability via eudaimonic well-being to personal success was more negative, which aligns with the idea that entrepreneurs without online business activities feel more strain and less success. However, no difference between the groups was found in terms of business success. This, again, may communicate that doing something is related to feeling more control and mastery, which is beneficial for well-being and perhaps inducing a feeling of being personally successful. For business success, however, there is no difference, and here, it may be that, at the time of the data collection in 2020, it was difficult to see how online business activities would develop over the crisis. It is also conceivable that for some entrepreneurs, starting an online business involved costs, and it was yet unknown to what extent that would attract new customers and entirely compensate for the loss of direct contact with customers. Concerning loneliness, a somewhat surprising finding was that the group engaging in online business activities had a more negative association between loneliness and personal success via eudaimonic well-being. Again, for business success this condition made no significant difference. Here, a potential explanation could be that engaging in online business was seen as a restriction from the normal in-person meetings with the clients, and felt more remote, accentuating the negative effects of loneliness. For the other group, being online may not have been an option, or not needed, so that this particular condition made no difference.

### Limitations

There are several limitations of the present study that are important to mention. The sample is based on convenience sampling, which undermines generalizability to the general population of entrepreneurs in Sweden. When the study took place at the beginning of the pandemic, businesses that were particularly affected were found in the hotel, restaurant and service sectors, in the consultancy sector (e.g., selling training and educational courses) and therapy (e.g., psychotherapy with face-to-face meetings in small rooms, massage or physiotherapy incompatible to distancing recommendations). Female business owners are overrepresented in these sectors. Additionally, Swedish women, more often than Swedish men, run smaller-scale businesses with lower financial assets (Braunerhjelm et al., 2019). These facts, together with the use of snowball tactics to collect responses, may explain the overrepresentation of women in our data. Thus, although not representative for all entrepreneurs in Sweden, the data may be more representative for those most affected by the pandemic. Their experiences are both valid and valuable and perhaps most relevant to study in order to enhance knowledge about how potential entrepreneurial stressors relate to strain and success, and to what extent these relations can be mitigated by entrepreneurial actions in a given context.

Another limitation concerns that the hypotheses were tested with cross-sectional data, which makes causality claims not possible. In other words, the study cannot demonstrate that eudaimonic well-being causally predicts personal and business success, although the relationships found in the data are well in line with the theoretical model. It is also worth noting that Dijkhuizen et al. (2018) tested for reverse effects and noted little evidence for a lagged association between business performance and well-being.

All data are self-reported data, which increases the risk that estimates for associations are inflated due to common method bias (CMV; Podsakoff et al., 2012). There have been substantial debates among scholars about the extent of the impact of CMV, with some suggesting that CMV may constitute an urban legend (Spector, 2006). Others calculated that monomethod research can produce more accurate estimates of relationships than other method approaches (Lance et al., 2010). To circumvent any potential bias of CMV, we employed several strategies for the study design (e.g., using different answer scales for measures, including negatively coded items, spreading out the study variables across the survey, guaranteeing confidentiality to participants). We also calculated the Harman test for common method variance, although this test has been heavily criticized (for discussion on the merits and shortcomings of this test, see Podsakoff et al., 2012), to further evaluate the risk that most variance is explained by a single factor which would signal a risk that CMV substantially affects the reported results. Different extraction approaches all provided more than one factor, and the first factor accounted for 32%, 17% and 15% of the total variance, respectively. Results of the Harman test provide little evidence of common method bias. Self-report measures are also most appropriate when the goal is to assess what participants subjectively perceive. In this study, most of the concepts of interest refer to perceptions of the entrepreneur, which are difficult to administer in an objective fashion. Particularly with regard to business success, one may have found different results when assessing the concept with objective data. For instance, it has been suspected that even when objective performance may be similar, entrepreneurs with lower well-being values may perceive less subjective business success (Gorgievski et al., 2010). Undoubtedly, eudaimonic well-being and personal fulfillment are conceptually closely related. However, although correlations between eudaimonic well-being and personal fulfillment were significant (*r* = .49, *p* < .001), additional confirmatory factor analyses supported the distinctiveness of the two concepts. We thus conclude that measurement error has likely not distorted results.

### Practical implications and final conclusions

Despite the limitations of this study, the results of the study do hint at several implications for researchers, policy-makers and entrepreneurs. For researchers, the external and global crisis of the pandemic is an important ground for research on how entrepreneurs move through the various stages of the pandemic and what the consequences are of the various countries’ approaches to supporting businesses. We see a great demand for further exploration of the effects of the COVID-19 pandemic long-term as well as the need to pay attention to contextual circumstances related to how countries or businesses were affected by and sought to tackle the crisis.

One may also position this article in the wider research area on business resilience – in small and resource-constrained businesses, the resilience of the business and of the individual entrepreneur are closely linked (Gianiodis et al., 2022). While not explicitly framed as resilience, this present study investigated business success and personal success, two aspects resembling firm resilience and entrepreneur resilience. Business and personal success correlated positively with one another, but correlations were not very strong and predictors differed. Gianiodis et al. (2022) asked for more research on moderating factors in order to provide practical guidance on how businesses and entrepreneurs may increase their resilience. Here, the present study contributes to these calls by investigating seeking government support and online business activities as moderating factors for predicting business and personal success.

Likewise, policy-makers can make use of the results that the application for government support schemes was not helping entrepreneurs in reducing the negative effects of unpredictability or loneliness. We suspect that entrepreneurs having applied to some support scheme were unsure of whether they would get the support and also gave away their agency as soon as they submitted their application. Instead, it may have been a more supportive process if the guidelines and the application forms were clear, so that applying would be equivalent to receiving or at least better communicating the chances of approval against pre-specified criteria that entrepreneurs could check in advance. Likewise, to buffer the negative effects of loneliness, it may have been more helpful if the government support schemes were made more personal, such that a direct line of communication between government agency and entrepreneur could be established. As entrepreneurs lack the organizational safety net that employees have, it might be relevant for policy-makers to establish equivalent forms of safety net for entrepreneurs, at least during a crisis of this magnitude. In the aftermath of the crisis, studies such as this one may be of help to evaluate the effectiveness of government support given. Our study emphasizes that such evaluations need to include not only business-related outcomes, but also outcomes in terms of well-being and personal success and fulfillment. This seems to be warranted especially if the goal is to encourage a higher percentage of the population to engage in entrepreneurship that creates job opportunities. In an external crisis that threatened the livelihood of many entrepreneurs, policies are needed that truly make entrepreneurial actions to mitigate stress likely to succeed, otherwise it is difficult to portray entrepreneurship as an attractive and sustainable career option for the future.

For entrepreneurs, the results of this study emphasize the relevance of well-being as a resource that helps facilitate business and personal success. As we studied eudaimonic well-being, this study also highlights that this more active dimension of well-being might be particularly relevant to foster, through creating purpose, growth and mastery experiences. Another less positive implication is based on the results that engaging in online business activities increased the negative effects of loneliness. While digitalization is seen as an important development for a future readiness of businesses (Tillväxtverket, 2018), the present study’s results indicate a worrying sign that engaging with online business is not a panacea to achieve better well-being and success.
